# Physiological analyses of swallowing changes due to chronic obstructive pulmonary disease in anesthetized male rats

**DOI:** 10.3389/fphys.2024.1445336

**Published:** 2024-08-07

**Authors:** Kouta Nagoya, Takanori Tsujimura, Midori Yoshihara, Masahiro Watanabe, Jin Magara, Katsushige Kawasaki, Makoto Inoue

**Affiliations:** ^1^ Division of Oral Functional Rehabilitation Medicine, Department of Oral Health Management, Showa University School of Dentistry, Tokyo, Japan; ^2^ Division of Dysphagia Rehabilitation, Niigata University Graduate School of Medical and Dental Sciences, Niigata, Japan; ^3^ Department of Hygiene and Oral Health, Showa University School of Dentistry, Tokyo, Japan; ^4^ Division of Oral Anatomy, Niigata University Graduate School of Medical and Dental Sciences, Niigata, Japan

**Keywords:** chronic obstructive pulmonary disease (COPD), respiration, swallowing, electromyography, rat

## Abstract

Chronic obstructive pulmonary disease (COPD) was previously known as chronic bronchitis and emphysema. It has various main symptoms, such as dyspnea, chronic cough, and sputum, and is often accompanied by dysphagia. Although many published clinical reports have described COPD-related dysphagia, the physiological mechanisms underlying swallowing changes due to COPD remain unclear. Therefore, we analyzed how COPD affects the swallowing reflex using COPD model rats. We performed an electrophysiological study of respiration and swallowing using COPD model induced by intratracheal administration of porcine pancreatic elastase and lipopolysaccharide in Sprague-Dawley male rats. To identify the respiration and swallowing responses, electromyographic activity was recorded from the diaphragm, digastric (Dig), and thyrohyoid (TH) muscles. We confirmed COPD using micro-computed tomography analysis and hematoxylin and eosin staining of the lungs. The duty cycle was defined as the ratio of the inspiration duration to the total respiratory duration. In COPD model rats, the duty cycle was significantly higher than that in control rats. The frequency of the swallowing reflex evoked by electrical stimulation of the superior laryngeal nerve during the inspiration phase was higher in COPD model rats than in control rats. Furthermore, long-term COPD altered Dig and TH muscle activity without pathological muscle change. Our results suggest that COPD increases the frequency of swallowing initiation during the inspiration phase. Furthermore, long-term COPD affects swallowing-related muscle activity without pathological muscle changes. These physiological changes may increase the risk of developing dysphagia. Further studies are necessary to clarify the mechanisms contributing to the functional changes in respiration and swallowing in COPD.

## 1 Introduction

Pneumonia is a leading cause of hospitalization and mortality in older patients (Loeb et al., 1999; Kaplan et al., 2002). In particular, the incidence of aspiration pneumonia accounts for >80% of pneumonia cases among older persons (Teramoto et al., 2008). The major causes of aspiration pneumonia are normal oral and/or pharyngeal flora, which may result from dysphagia in many cases (Yamanda et al., 2010).

The prevalence of dysphagia may vary among medical diseases such as neurological (Panebianco et al., 2020), cerebrovascular (Smithard, 2016), neuromuscular diseases (Argov and de Visser, 2021), and head and neck tumors (Salama et al., 2008). Respiratory diseases also pose a risk of dysphagia (Ghannouchi et al., 2016). Chronic obstructive pulmonary disease (COPD) is a major chronic respiratory disease associated with dysphagia (Lin and Shune, 2020).

Although dyspnea on exertion, chronic cough, and sputum are the main symptoms of COPD (Ishii and Kida, 2014), it is no longer considered a disease that affects only the lungs. COPD induces muscle weakness, cardiovascular events, and metabolic syndrome (Fabbri et al., 2008). In addition, patients with COPD sometimes experience deteriorating activities of daily living (ADL) due to emaciation and malnutrition (Weekes et al., 2009). We should realize that many organs are involved in the neuromuscular system of respiratory and swallowing functions (Mckeown et al., 2002; Fregosi and Ludlow, 2014); therefore, COPD affects not only breathing but also eating behaviors.

Characteristics of dysphagia due to COPD include discoordination between respiration and swallowing, delayed swallowing reflex, poor laryngeal elevation, and poor upper esophageal sphincter opening (Mokhlesi et al., 2001; 2002). Although several clinical reports have described COPD-related dysphagia (Stein et al., 1990), the physiological changes in swallowing that accompany COPD remain unknown. In this study, we investigated how COPD contributes to changes in respiration and swallowing using COPD model rats. We hypothesized that COPD remarkably changes respiratory function but does not affect swallowing motor action, which may, in turn, alter the coordination of respiration and swallowing. In human with COPD, the changes in breathing are primarily observed. The changes in swallowing, such as poor laryngeal elevation and poor upper esophageal sphincter opening, have been observed; however, no studies have reported the changes in swallowing related muscle activity, and we thought that the swallowing motor action itself does not change.

## 2 Materials and methods

### 2.1 Ethical approval

This study was reviewed and approved by the Niigata University Intramural Animal Care and Use Committee (SA00616) and was performed in accordance with the Guiding Principles for the Care and Use of Laboratory Animals (National Institutes of Health, Bethesda, MD, United States).

### 2.2 Animals

All experiments were performed on 80 male Sprague-Dawley rats (300–350 g; Charles River Laboratories, Yokohama, Japan). We housed three rats in each cage in the vivarium, which was maintained at a controlled temperature (23.5°C) with 12 h light/dark cycles. The rats had *ad libitum* access to food and water.

### 2.3 Induction to COPD

**FIGURE 1:**
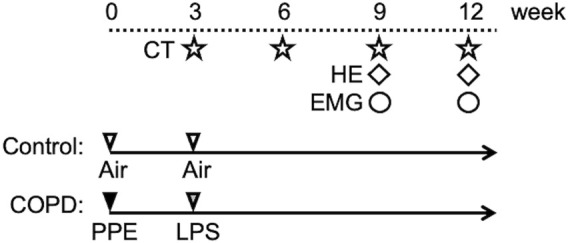
Experimental protocol. On the first day of the experiment, PPE was administered intratracheally to Sprague-Dawley rats. After 3 weeks, LPS was administered using the same method. At weeks 3, 6, 9, and 12, all rats were scanned using micro-CT. Hematoxylin and eosin (HE) staining of the lung was performed in the 9th week. HE staining of swallowing related muscles was performed in the 12th week. We gained the electromyographic (EMG) data in the 9th and 12th weeks.

We established COPD model rats using a method modified from a previous study (Kobayashi et al., 2013). The animals were anesthetized with an intraperitoneal injection of midazolam (2.00 mg/kg), medetomidine (0.375 mg/kg), and butorphanol tartrate (2.50 mg/kg) (MMB) before instilling porcine pancreatic elastase (PPE; FUJIFILM Wako Pure Chemical Corporation Ltd., Osaka, Japan). Twenty-eight U/100 g of PPE dissolved in 100 μL phosphate-buffered saline (PBS) was intratracheally administered using a MicroSprayer drug delivery device (Natsume Seisakusho Ltd., Tokyo, Japan). Three weeks later, lipopolysaccharide (LPS; Shigma-Aldrich Co., St. Louis, United States) dissolved in 100 μL of PBS was intratracheally administered (5 mg/mL). To confirm enhanced lung inflammation and the progression of emphysema, we evaluated the lungs using micro-computed tomography (CT) and hematoxylin and eosin (HE) staining. In contrast, the control rats were produced by administering the same amount of air at the same point for PPE and LPS. Figure 1 shows the time course of the experiments.

The body weight (BW) of the COPD model and control rats was measured simultaneously using micro-CT scanning. To compare BW changes between the COPD model and control rats, we calculated the normalized BW to that measured on the day of PPE injection (day 0).

### 2.4 Micro-CT analysis

Rats were anesthetized with MMB, and their lungs were scanned using a high-resolution microscanner, the CosmoScan GX (Rigaku Corporation, Tokyo, Japan). Additionally, micro-CT was run with an isometric resolution of 72 μm; the X-energy was set at 90 KV and 88 μA with a pixel size of 50 μm, an exposure time of 4 min (Katagiri et al., 2021). Image acquisition was respiratory-gated. Micro-CT scanning was calibrated according to the manufacturer’s protocol, and CosmoScan GX software was used to reconstruct the three-dimensional image. From the data obtained from micro-CT scanning, we calculated the percentage of the low-attenuation area (LAA%) as the ratio of the LAA to the total lung area (Sasaki et al., 2015). The LAA represents individual areas of emphysematous destruction. The LAA thresholds were determined in the range of −400 to −700 Hounsfield units (HU) in our study. LAA% indicates the proportion of emphysema to the total lung and can be used to evaluate the severity of COPD (Sun et al., 2017). Micro-CT images were obtained every 3 weeks after PPE administration until the 12th week.

### 2.5 Histological analysis

Nine COPD models and eight control rats were used for the histological study. Following sacrifice and perfusion with a 10% formalin neutral buffer solution (FUJIFILM Wako Pure Chemical Corporation Ltd., Osaka, Japan), the bilateral lung and swallowing-related muscles, left digastric (Dig) and thyrohyoid (TH), were rapidly isolated and fixed in it. The fixed lungs were stained with HE in the 9th week after PPE administration, while swallowing-related muscles were stained in the 12th week after PPE administration (Feldman and Wolfe, 2014). All staining experiments were performed by Biopathology Institute Co., Ltd (Oita, Japan). Additionally, the number of muscle cells was compared using image analysis software (ImageJ, version 1.53; National Institutes of Health, United States) (Higashino et al., 2014).

### 2.6 Electrophysiological data acquisition and analysis

The rats were anesthetized with an intraperitoneal urethane injection (1.3 g/kg, N = 44), which was supplemented to maintain anesthesia at a level where neither the corneal reflex nor spontaneous eye movement occurred.

With the rat in the supine position, a midline incision was made along the ventral aspect from the pogonion to the caudal portion of the neck. All animals underwent tracheotomy before recording. A tracheotomy was performed at the level of the upper side of the trachea.

For electromyographic (EMG) recording of swallowing-related muscles, bipolar enamel-coated copper wire electrodes (0.23 mm in diameter and 2 mm in inter-polar distance) were inserted into the left Dig and TH. To assess the diaphragm (Dia) activation, pairs of enamel-coated copper wire electrodes were implanted (intramuscularly using an abdominal approach) into the mid-costal regions of the right side of the Dia. We determined that the respiratory cycle began at the onset of inspiration, which was defined as the onset of the Dia EMG burst, and ended at the next onset of the Dia EMG burst (Jimenez-Ruiz et al., 2018).

The EMG electrodes were connected to an amplifier (AM-601G; Nihon Kohden, Tokyo, Japan), and the recorded signals were amplified, high-pass filtered (50–100 Hz), and stored on a computer hard disk at a sampling rate of 10 kHz. All data were analyzed using the Spike2 analysis package (Cambridge Electronic Design, Cambridge, United Kingdom).

To evoke the swallowing reflex, bipolar enamel-coated silver wire electrodes (0.2 mm diameter) for applying electrical stimulation were placed on the right side of the superior laryngeal nerve (SLN), which was electrically stimulated repetitively (0.2 ms pulse duration; 30 Hz). The stimulation threshold for evoking the swallowing reflex was defined as the minimum stimulus intensity required to evoke swallowing at least once during SLN stimulation for 10 s. The current intensity was determined as 1.2 times and 2 times the stimulation threshold, and the swallowing reflex was identified by Dig and TH EMG bursts and laryngeal elevation.

The swallowing reflexes were evoked by 10 s repetitive SLN stimulation. The swallowing reflex responses were characterized by both Dig and TH EMG bursts. Using the second swallowing response, the EMG burst duration (defined as the time interval from onset to offset of Dig and TH EMG bursts), rising time (defined as the time interval from onset to peak of Dig and TH EMG bursts), and falling time (defined as the time interval from peak to offset of Dig and TH EMG bursts) were compared between the groups.

To evaluate the temporal relationship between the Dia and Dig EMG bursts, the time interval between the onset of the Dia and Dig EMG bursts was compared between the groups.

The timing of evoking swallowing reflex in respiratory cycle was analyzed using Dig EMG bursts immediately before swallowing.

We used the Dia EMG burst to analyze respiratory function. Inspiration duration was defined as the duration of Dia bursting. In contrast, expiration duration was defined as the duration of non-Dia bursting. From the five respiratory cycles in each animal, the mean duration of total respiration, inspiration, and expiration, as well as the respiratory rate and duty cycle, were calculated as the baseline. The duty cycle was calculated as the ratio of inspiration duration, defined as the duration of the Dia EMG burst to the total respiration duration (total duration of inspiration and expiration). Respiratory function was compared between COPD model and control rats.

To compare in a time-dependent change, we analyzed EMG recordings from the 9th and 12th weeks after PPE administration.

After EMG recording, the animals were euthanized by administration of an overdose of urethane.

### 2.7 Statistics

All statistical analyses were performed using SPSS version 26 (SPSS Japan, Tokyo, Japan). Data, except for BW, are reported as the mean ± standard error, and BW as the mean ± standard deviation. Figures 4E, F, 5B–D, 6C–E were shown as the box plot with interquartile range. Change in BW was compared using a Student’s t-test. The duration of inspiration and expiration in the control and COPD model rats was tested using a paired t-test. The respiration rate, duty cycle, duration (rising time, falling time, and total) of Dig and TH, and the interval from Dia to Dig were analyzed using one-way ANOVA, followed by the Bonferroni *post hoc* test. The respiration and EMG data were compared between control and 9th week and between control, 9th week and 12th week using parametric or nonparametric test. The difference of the swallowing reflex occurrence in the distribution between the control and COPD model was tested with a χ^2^ test. Statistical significance was set at *P* < 0.05.

## 3 Results

**FIGURE 2:**
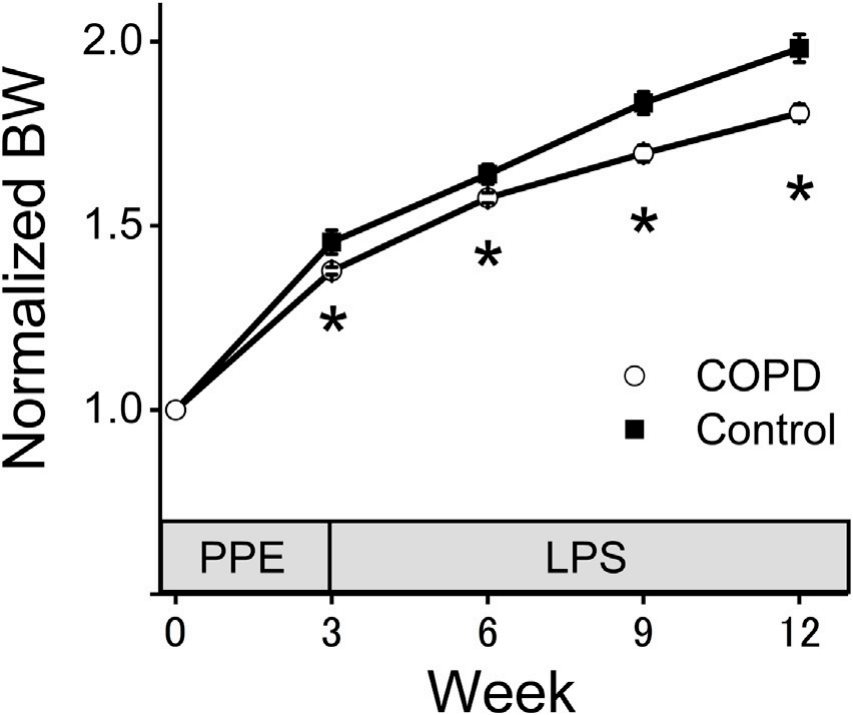
The normalized body weight (BW) of the COPD model rats was significantly lower than that of the control rats. The BW of the COPD model and control rats was measured every 3 weeks. The normalized BW was a ratio compared with BW at the time of porcine pancreatic elastase injection. *P < 0.05.

Figure 2 shows the changes in normalized BW. There was no significant difference of the BW between control and COPD rats at the start of measurement. The normalized BW of the COPD model rats was significantly lower than that of the control rats (1.38 vs. 1.46 at 3rd week; *P* = 0.036, 1.58 vs. 1.64 at 6th week; *P* = 0.040, 1.70 vs. 1.83 at 9th week; *P* = 0.019, 1.81 vs. 1.98 at 12th week; *P* = 0.023).

### 3.1 Evaluation of COPD model rats

To confirm enhanced lung inflammation and the progression of emphysema, we evaluated COPD using micro-CT images and HE staining.

**FIGURE 3:**
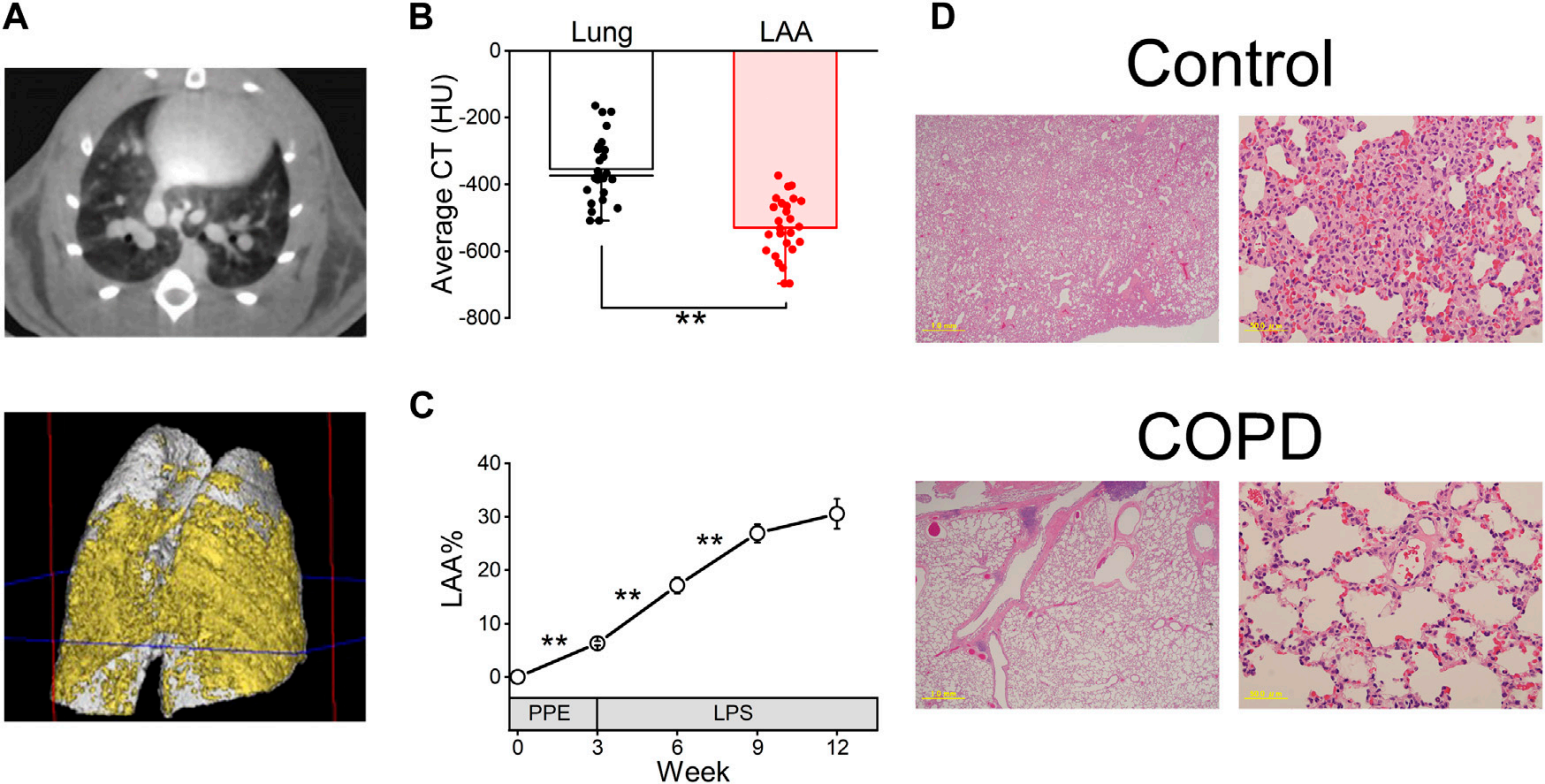
COPD was confirmed using micro-computed tomography (CT) analysis and hematoxylin and eosin (HE) staining. **(A)**, representative transverse micro-CT image (upper panel) and three-dimensional reconstructed image (lower panel, gray; whole lung, yellow; emphysema) of the COPD model rat at the 9th week after porcine pancreatic elastase administration. **(B)**, the low attenuation area (LAA) thresholds range from −400 to −700 Hounsfield units in our study. **(C)**, LAA% of COPD model rats was significantly increased until the 9th week compared with the first day, 3rd week and 6th week. **(D)**, HE-stained images of the control and COPD model rats at the 9th week. **P < 0.01.

As shown in Figure 3A, the transverse micro-CT image of COPD model rats in the 9th week after PPE administration clearly indicated emphysema as a black area. The three-dimensional reconstructed image of the COPD rat lung showed that both sides of the lung exhibited widespread emphysema (yellow).

The average CT value of LAA% is significantly lower than that of the lung (−528.65 ± 17.38 HU vs. −354.57 ± 19.46 HU, *P* < 0.01, Figure 3B). While the LAA% of the COPD model rats significantly increased over time until 9th week (6.32% ± 0.40% at 3rd week, 17.13% ± 1.48% at 6th week and 26.89% ± 1.72% at 9th week, Figure 3C), it was not significantly different between the 9th and 12th weeks after PPE administration (30.56% ± 2.80% at 12th week).

Furthermore, HE staining was performed in the 9th week after PPE administration. Figure 3D shows representative lung sections from the control and COPD model rats. The COPD model rats showed enlarged alveolar spaces and destroyed alveolar walls.

These results indicated that the emphysema of lung progressed over a period of time in COPD model rats.

### 3.2 Respiratory changes in COPD model rats

**FIGURE 4:**
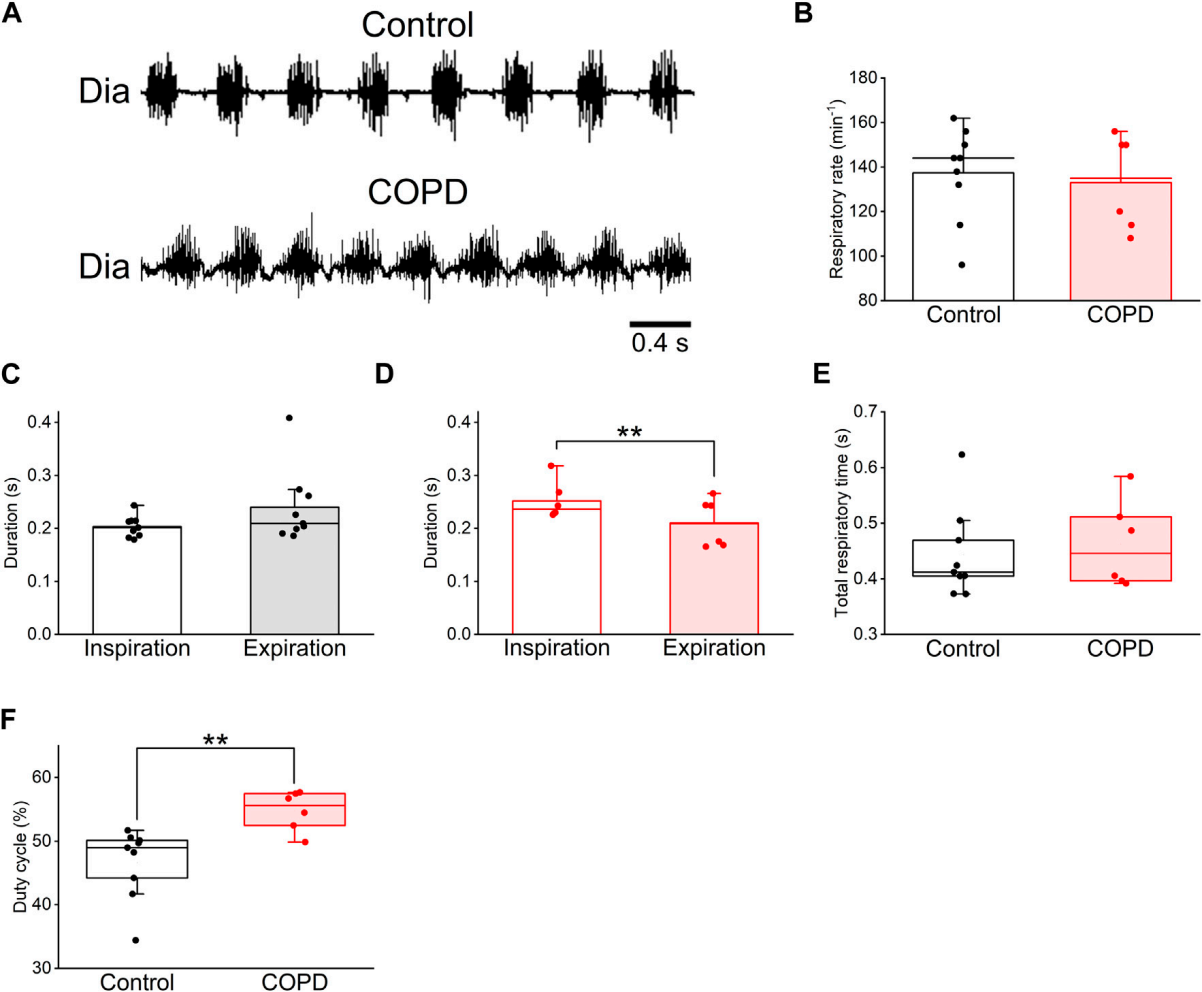
The duty cycle of the COPD model rats was significantly higher than that of the control rats. **(A)**, typical diaphragm electromyographic traces of eupnea in the control (upper) and COPD model (lower) rats. **(B)**, the respiratory rate of the COPD model rats was not significantly different from that of control rats. **(C,D)**, the mean duration of inspiration and expiration of the control **(C)** and COPD model **(D)** rats, respectively. The inspiratory duration of the COPD model rats was significantly longer than their expiratory duration. **(E)**, the total respiratory duration of the COPD model rats was not significantly different from that of control rats. **(F)**, the duty cycle of the COPD model rats was significantly higher than that of the control rats. **P < 0.01.

To analyze changes in respiration and swallowing due to COPD, we used the COPD model rats in the 9th week after PPE administration because the LAA% change in the 9th week was sufficient for analysis and comparison with control rats. First, we investigated the respiratory changes in the COPD model rats and compared them with those in the control rats using Dia EMG recordings. The respiratory rate of the COPD model rats did not significantly differ from that of the control rats (133 ± 7.9 cycles/min vs. 137 ± 6.6 cycles/min, *P* = 0.705, Figures 4A, B). Although the mean duration of inspiration did not show a significant difference compared to that of expiration in control rats (0.202 ± 0.006 s vs 0.244 ± 0.020 s, *P* = 0.137, Figure 4C), the mean duration of inspiration of the COPD model rats was significantly longer than that of expiration 0.252 ± 0.013 s vs 0.210 ± 0.017 s, *P* = 0.009, Figure 4D). The total respiratory duration also did not show a significant difference between the control and COPD model rats (0.446 ± 0.023 s vs 0.462 ± 0.029 s, *P* = 0.776, Figure 4E). Eventually, the duty cycle of COPD model rats was significantly higher than that of control rats (54.78% ± 1.17% vs. 46.63% ± 1.76%, *P* = 0.009, Figure 4F).

### 3.3 Changes in coordination between respiration and swallowing in COPD model rats

**FIGURE 5:**
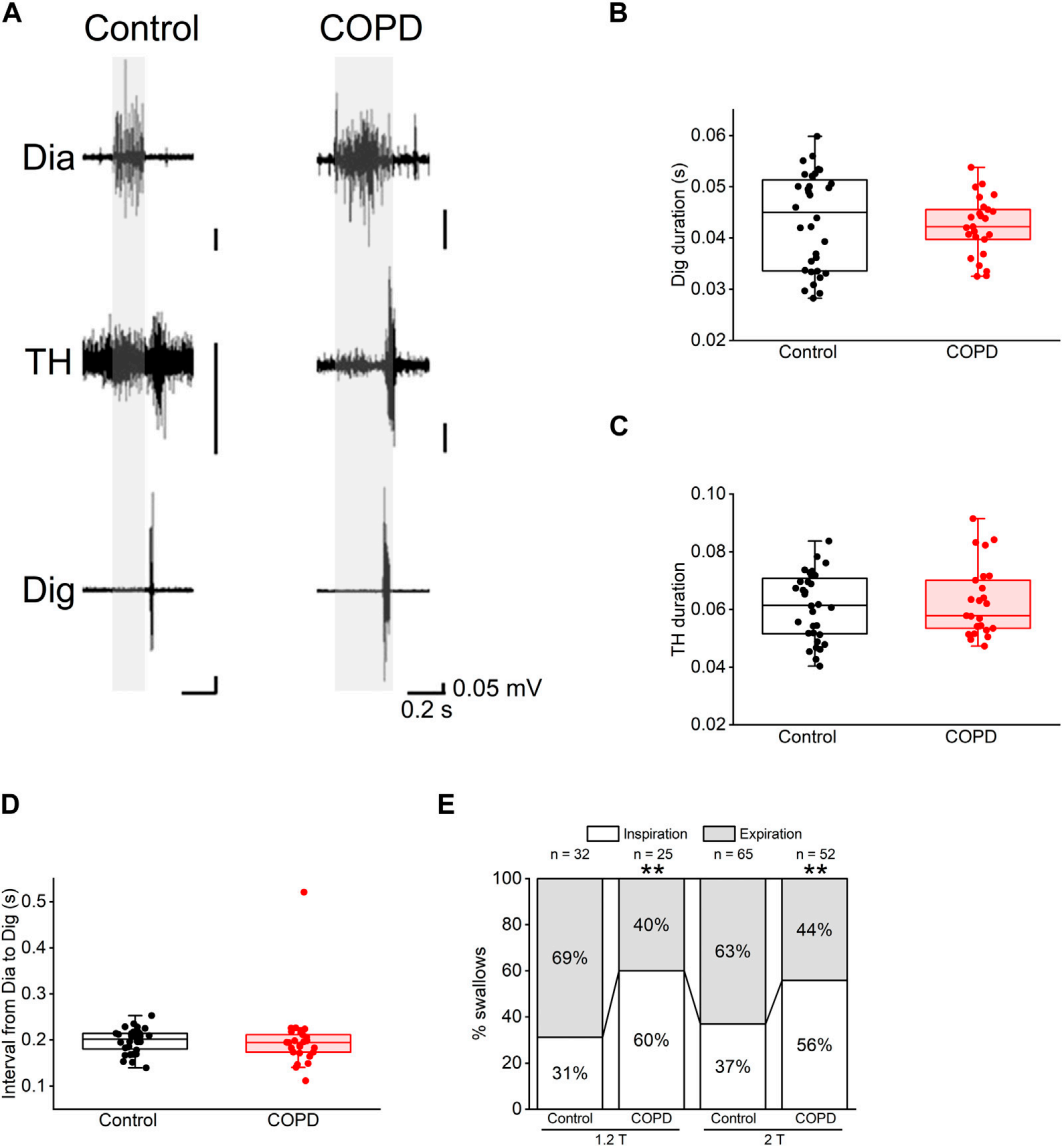
Most of the swallowing reflex in COPD model rats occurred in the inspiratory phase. **(A)**, representative electromyographic traces of the swallowing reflex in the control (left) and COPD model (right) rats. **(B,C)**, the durations of the Dig **(B)** and TH **(C)** did not show significant differences between the control and COPD model rats. **(D)**, the interval from inspiration to the start of the Dig activity did not show a significant difference between the control and COPD model rats. **(E)**, stacked bar chart showing the percentages of the swallowing reflex in the inspiratory (white column) and expiratory phases (black column) in the control and COPD model rats. Dig: digastric TH: thyrohyoid **P < 0.01.

We analyzed the EMG activity to explore the effect of COPD on the coordination between respiration and swallowing. Figure 5A shows representative EMG traces of Dia, TH, and Dig in the control and COPD model rats. Dia EMG bursts were observed in the inspiratory phase and were inhibited during swallowing, particularly in the control rats. There was no significant difference in the duration of the Dig and TH EMG bursts between the COPD model and control rats (0.042 ± 0.001 s vs. 0.043 ± 0.002 s for Dig, 0.063 ± 0.002 s vs. 0.061 ± 0.002 s for TH, *P* = 1.000 for both Figures 5B, C). Furthermore, the rising and falling times of these bursts did not significantly differ between the COPD model and control rats (rising time, 0.019 ± 0.001 s vs. 0.018 ± 0.001 s for Dig, *P* = 0.823, 0.030 ± 0.003 s vs. 0.032 ± 0.002 s for TH, *P* = 0.174; falling time, 0.023 ± 0.002 s vs. 0.025 ± 0.001 s for Dig, *P* = 0.829, 0.033 ± 0.002 s vs. 0.029 ± 0.002 s for TH, *P* = 0.613).

Regarding the temporal relationship of onset time between these EMG bursts, there was no difference in the time interval from the onset of Dia EMG burst to that of the Dig EMG burst between COPD and control rats (0.200 ± 0.015 s vs. 0.198 ± 0.005 s, *P* = 0.470, Figure 5D). However, the frequency of swallowing initiation during the inspiratory phase in the COPD model rats was significantly higher than that in control rats (60.00% vs. 31.25%, 1.2 times the stimulation threshold, *P* < 0.01, Figure 5E). This was also the case at two times the stimulation threshold, where the results showed a similar trend (55.77% vs. 36.92%, *P* < 0.01, Figure 5E). These results suggest that the modulation of temporal relationships is attributable to changes in Dia EMG bursts.

### 3.4 Time-dependent changes in COPD model rats

**FIGURE 6:**
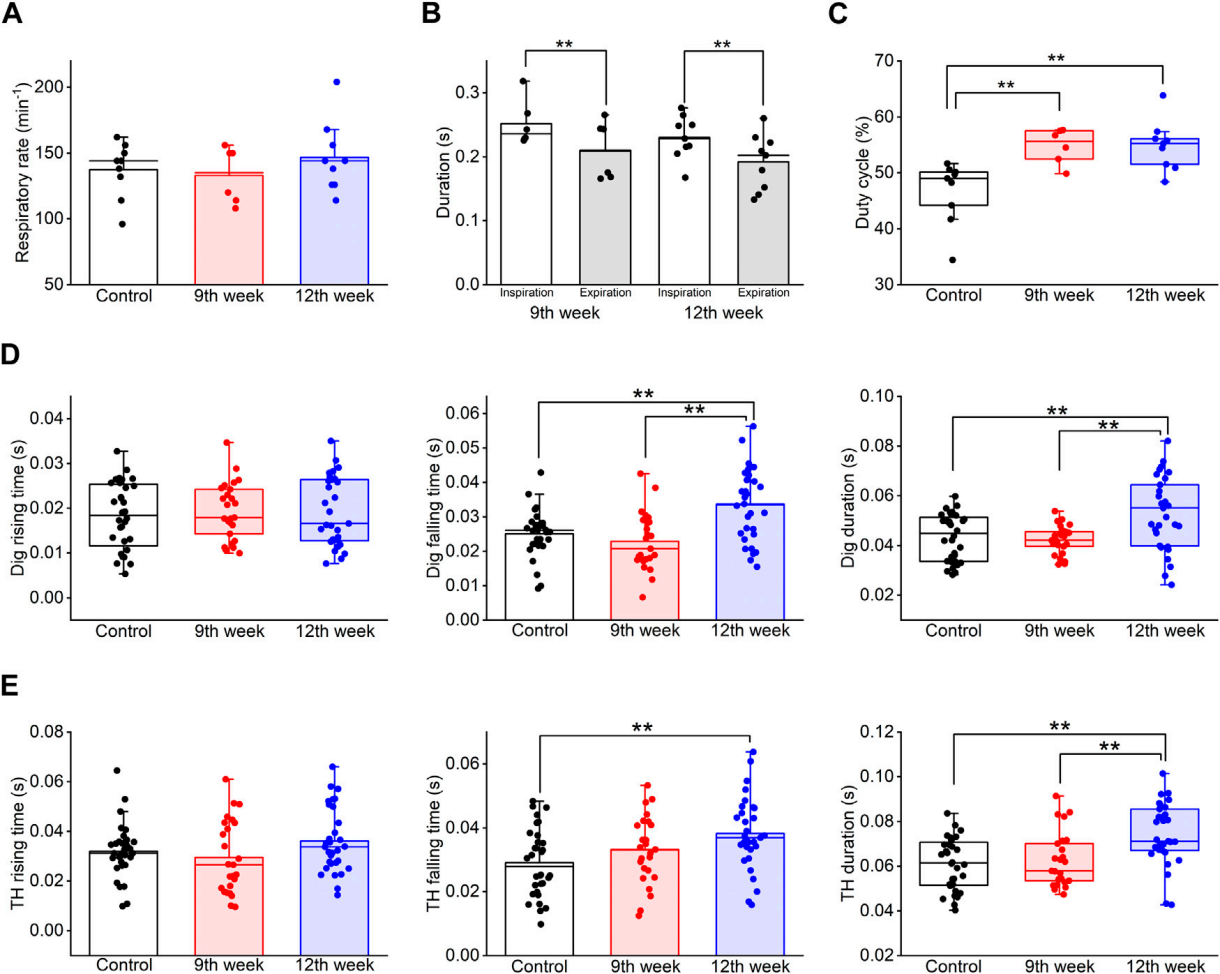
Long-term COPD affected swallowing-related muscle activity. **(A–C)**, the respiration rate **(A)**, the duration of inspiration and expiration **(B)**, and the duty cycle **(C)** of the COPD model rats at the 12th week after porcine pancreatic elastase administration did not significantly differ from those at the 9th week. **(D)**, the falling time and the duration of the Dig in COPD model rats at the 12th week was significantly longer than that in the control rats (falling time: 133%, duration: 122%). **(E)**, the falling time and the duration of the TH in COPD model rats at the 12th week was significantly longer than that in the control rats (falling time: 131%, duration: 122%). Dig: digastric TH: thyrohyoid *P < 0.05, **P < 0.01.

We analyzed changes in the COPD model over time. The LAA% of the COPD model rats at the 12th week after PPE administration did not show a significant change compared to that at the 9th week (26.89% ± 1.72% at 9th week vs. 30.56% ± 2.80% at 12th week, *P* = 1.000, Figure 3C). The mean respiratory rate of the COPD model rats at the 12th week after PPE administration tended to be high compared with that at the 9th week and that of the control rats (137.3 ± 6.6 cycles/min in control vs. 133.0 ± 7.9 cycles/min at 9th week vs. 146.7 ± 8.5 cycles/min at 12th week, *P* = 0.499, Figure 6A). At the 9th and 12th weeks, the mean duration of inspiration was significantly longer than that of expiration (0.252 ± 0.013 s vs. 0.210 ± 0.016 s at 9th week, 0.230 ± 0.011 s vs. 0.192 ± 0.014 s at 12th week, *P* = 0.01, Figure 6B). Additionally, the duty cycle of the COPD model rats at the 9th and 12th weeks after PPE administration was significantly higher than that of control rats (46.63% ± 1.76% for control, 54.78% ± 1.17% at 9th week, 54.83% ± 1.39% at 12th week, *P* = 0.003). However, it did not significantly differ between the 9th and 12th week (*P* = 1.000, Figure 6C).

Although the frequency of swallowing initiation during the inspiratory phase in the COPD model rats at the 12th week tended to be higher than that of the control rats, there was no significant difference compared to that of the COPD model rats at the 9th week (61.29% for 12th vs. 31.25% for control vs. 60% for 9th).

Finally, the Dig and TH EMG burst properties were compared among the time periods. The falling time of the Dig and TH EMG bursts at the 12th week after PPE administration was significantly longer than that of the control rats (0.033 ± 0.002 s at 12th week vs 0.025 ± 0.001 s at control for Dig, *P* = 0.01, 0.038 ± 0.002 s at 12th week vs 0.029 ± 0.002 s at control for TH, *P* = 0.006, Figures 6D, E). Furthermore, the Dig and TH EMG duration in the COPD model rats at the 12th week showed a significant difference compared to that of the control and the measurements taken at the 9th week (0.053 ± 0.003 s at 12th week vs 0.043 ± 0.002 s at control vs 0.042 ± 0.001 s at 9th week for Dig, *P* = 0.005, 0.074 ± 0.002 s at 12th week vs 0.061 ± 0.002 s at control vs 0.062 ± 0.003 s at 9th week for TH, *P* = 0.001, Figures 6D, E).

### 3.5 Muscle changes in COPD model rats

**FIGURE 7:**
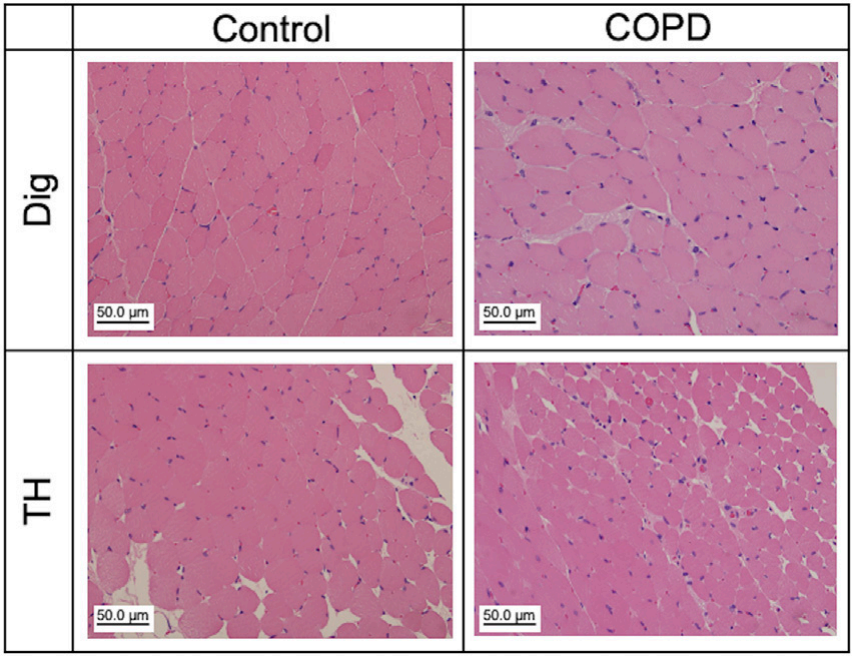
Muscle atrophy and other pathological abnormalities were not confirmed in Dig and TH muscles in the COPD model rats. Representative hematoxylin and eosin staining images of the Dig (upper) and TH (lower) muscles in the control (left) and COPD model (right) rats. Dig: digastric TH: thyrohyoid.

We analyzed the changes in swallowing-related muscular cells at the 12th week after PPE administration. Figure 7 shows representative HE-stained images of the Dig and TH. The control and COPD rats did not differ in the mean muscle cell numbers of Dig and TH (93.50 vs 145.70 for Dig, 264.00 vs 175.67 for TH, respectively, N = 2–3). We confirmed the alternation of the myonuclei in COPD model rats, but neither muscle image showed any another pathological changes. More detailed analysis is needed, including the changes fiber cross-sectional areas in the future study.

## 4 Discussion

In this study, we investigated the effects of COPD on respiration and swallowing in rats. Our study clearly revealed that COPD changed the respiratory cycles with significant changes in swallowing muscle activity. Specifically, the duty cycle increased, and swallowing initiation was dominant during the inspiratory phase.

### 4.1 COPD model rats

COPD model animals are most commonly produced by exposing them to cigarette smoke (Ghorani et al., 2017). Using this method, a variety of animal species, such as mice, rats, guinea pigs, dogs, and monkeys, can be used to mimic COPD (Jones et al., 2017). However, this method is complicated; thus, it takes a long time to produce COPD model animals. Li et al. (2012) reported that to produce the COPD model in rats, they had to follow a two-step treatment process. First, the rats were exposed to the smoke of eight cigarettes per treatment twice a day during the first 2 weeks. Then, from the 3rd to the 12th week, the rats were exposed to the smoke of 15 cigarettes per treatment, three times a day. In contrast, Wang et al., 2014 showed that it takes 4 months in a one-step process to establish a COPD rat model. In both cases, this process takes a long time to complete. Furthermore, rats are a poor model of COPD because they are relatively resistant to COPD compared to mice (March et al., 1999; Wright et al., 2008). Therefore, in this study, we generated a COPD rat model using the PPE-LPS combination method. The best advantage of this method is that COPD model rats can mimic exacerbations similar to the clinical situation. Kobayashi et al. established this combination method in mice and demonstrated that the LAA% of COPD model mice at 9 weeks after PPE administration was >40%. In our study, the LAA% of COPD model rats gradually increased up to 9 weeks after PPE administration, and at the 12th week, approximately 30% of the whole lung was emphysematous. We speculate that this difference in %LAA was due to the reagent dose. We modified the dose of intratracheal LPS administration of guinea pigs because the BW of the rats in our study was similar to that of guinea pigs (Pera et al., 2011). Furthermore, Mikami et al., 1989 demonstrated that the drug sensitivity of the guinea pig trachea was higher than that of other species. Therefore, although the %LAA reached 30%, the %LAA in our study was different from previous reports. The LAA% in human is varied depending on the severity of the disease. The Goddard score, which we evaluate using the LAA%, is evaluated by the total points for six sites of lungs (Goddard et al., 1982). Although we used rats for experiments, the results of this study show that the LAA% (about 30%) is two points out of five in the Goddard score, which may be a low level of severity. It is necessary to verify in the future study whether the disease worsens with an extended period of illness in this model.

We used P 15–18-week rats to obtain the data in this study. The age of these rats is equivalent to 40–50 years in humans, and they are not aged model rats. In humans, most patients with COPD are over 60 years of age. Therefore, it is possible that the swallowing function is already declining due to aging, and that the swallowing dynamics may worsen further as COPD becomes more severe. In this study, although the analysis of the lung by micro-CT and HE staining confirmed the COPD condition based on previous studies of COPD model animals, it is possible that the rats did not develop a fulminant condition and had only functional emphysema. In the future study, we need to verify using aged model rats.

### 4.2 Respiratory changes

A previous animal study used urethane as a general anesthetic in acute experiments to observe respiration and swallowing (Tsujimura et al., 2019). Urethane, at anesthetic doses, has been reported to have less of an effect on the resting respiratory function in rats (Sapru and Krieger, 1979; Maggi and Meli, 1986). Boon and Milsom suggested that the resting ventilation levels of anesthetized rats using urethane were similar to those of conscious rats. A low level of urethane does not cause significant respiratory depression or a reduction in sensitivity to hypoxia or hypercapnia (Boon et al., 2004). In our study, the dose of urethane used was 1.3 g/kg and was categorized as relatively low. Therefore, the respiratory changes observed in this study were not affected by urethane.

To assess respiratory activity, the duty cycle is commonly utilized as a parameter, defined as the ratio of the duration of inspiration to the total respiration duration (Jimenez-Ruiz et al., 2018). Depending on the animal condition (conscious/anesthetized), the duty cycle may vary widely. In conscious rats, the duty cycle is approximately 30%–74% (Kanbar et al., 2010; Mantilla et al., 2011; Rojas-Líbano et al., 2014; Jimenez-Ruiz et al., 2018). In anesthetized rats, it is dependent on the anesthetic used and is 35%–46% (Wasserman et al., 2000; Boon and Milsom, 2010; Jimenez-Ruiz et al., 2018). In our study, the mean duty cycle in control rats was 46.63% (ranging from 34.43% to 51.68%). Thus, we suggest that the respiratory condition of our control rats was comparable to that reported in previous studies. However, in our study, the duty cycle of the COPD model rats was higher than that of the control rats. In humans, COPD is commonly characterized by shortness of breath and an increase in expiration duration (Gorini et al., 1990), indicating a low duty cycle. Therefore, our results are inconsistent with those of previous studies on human COPD. One possibility for this discrepancy may be the use of anesthesia. Although urethane anesthesia does not change respiratory activity, it might prevent rats from voluntarily controlling their breathing. Thus, the COPD model rats could not exhale voluntarily, leading to a higher duty cycle in COPD model rats. This consideration is supported by the finding that the respiratory rate was not significantly different between the control and COPD model rats. Disease exacerbation is another factor that should be considered. Gorini M et al. reported that inspiratory and expiratory durations were significantly shorter during disease exacerbation in humans. This result indicates that the more severe the disease exacerbation, the smaller the duty cycle. Therefore, we considered that the COPD condition in the present study was not damaged as breathing changed. However, the CPOD rats exhibited a longer inspiration duration than that of the control rats due to the emphysema. Consequently, the duty cycle increased.

We produced the COPD model rats using the PPE and LPS combination method, and the infusion of LPS can acutely exacerbate COPD, where the mechanical stress in the alveolar wall (similar to raised intrathoracic pressure) may progress the emphysema owing to the weak alveolar wall destruction caused by the strong power. This may affect mechanosensitive input from the lungs to the respiratory center of the CNS. Hence, further studies in conscious conditions are necessary to understand not only control rats but also COPD.

### 4.3 Swallowing changes

We demonstrated that the swallowing reflex in the COPD model rats was predominantly evoked during the inspiration phase. Additionally, it has been reported that in most mammals (except humans), most of the swallowing reflex occurs during the inspiratory phase (Doty and Bosma, 1956; Kawasaki et al., 1964; McFarland and Lund, 1993). However, a few studies have demonstrated that the swallowing reflex is evoked during the expiratory phase in anesthetized cats (Doty and Bosma, 1956; Dick et al., 1993). Furthermore, recent studies reported that >90% of the swallowing reflex occurs during the expiratory phase in normal rats (Ghannouchi et al., 2013; Ouahchi et al., 2019; Ghannouchi et al., 2020), whereas in the present study, approximately 70% of the reflexes in control rats were evoked during the expiratory phase. This may be due to differences in the methods used to evaluate the respiratory phase. Although many studies measured respiratory variables using whole-body barometric plethysmography, we evaluated the respiratory phase using a Dia EMG burst. Therefore, we may not have been able to evaluate the respiratory phase in detail. In our study, the swallowing reflex was predominantly evoked during the inspiratory phase in the COPD model rats. Since the time interval from the onset of inspiration to that of swallowing was not different between the COPD model and control rats, and the duty cycle was larger in the COPD model than in the control rats, the change in the timing of swallowing initiation was due to changes in respiratory function, not the discoordination of respiration and swallowing. This is supported by previous clinical studies where patients with COPD were more likely to swallow a bolus during inhalation (Shaker et al., 1992; Gross et al., 2009; Cvejic et al., 2011). Therefore, COPD may not change the neural activity in respiratory and swallowing function coordination; however, it modulates respiratory neural activity, thus increasing the risk of bolus aspiration.

### 4.4 Long-term changes in respiration and swallowing

In this study, long-term COPD affected the EMG activity of the Dig and TH muscles. Muscle atrophy, muscle fiber type changes, and muscle fatigue are factors that extend muscle EMG duration (Arendt-Nielsen et al., 1989; Kupa et al., 1995; Cruz-Martínez et al., 2000). HE staining of Dig and TH revealed no muscle atrophy or other pathological features in the COPD model rats. Thus, we considered that changes in muscle fiber type and fatigue may be related to this phenomenon. Sarcopenia is a known factor that induces changes in muscle fiber type and fatigue (Rosenberg, 1997). Sarcopenia of the swallowing-related muscles remains unclear. In general, it is difficult to induce sarcopenia in swallowing-related muscles because they constantly receive inputs from the respiratory center of the CNS. However, Fogarty et al., 2019 reported that Dia sarcopenia is characterized by muscle fiber type changes, although the muscle receives inputs from the respiratory center of the CNS and is constantly activated. Thus, it is necessary to investigate the muscle fiber types and muscle force changes that mimic fatigue in the COPD model rats. Another possibility is that mechanosensitive input from the lungs to the respiratory center of the CNS may alter the neural activity in the respiratory center, leading to changes in muscle activity in the periphery. Previous studies have demonstrated that the Hering-Breuer reflex is diminished in patients with COPD (Tryfon et al., 2001). Furthermore, sleep apnea in adults and/or sudden infant death syndrome have also been suggested to be associated with dysfunctions in sensory neurons innervating the upper airway, including the oral and nasal cavities and larynx (Nonomura et al., 2017). Thus, it is estimated that the CNS and peripheral nervous system modify neural activity through mechanosensitive changes in the lungs. To clarify the connection between these changes and muscle activities, we must attempt to perform the experiment at the CNS level in a further study.

### 4.5 Limitation

This study has some limitations. First, the method of evoking the swallowing reflex involves SLN stimulation. It has been reported that SLN stimulation modulates phrenic nerve discharge and may change respiratory activity; therefore, it may be better to induce more natural swallowing, similar to that with water (Sun et al., 2011). However, the amount of bolus is related with swallowing induction, and in patients with COPD, a delay in inducing the swallowing reflex is recognized as dysphagia. Therefore, in this study, we used the SLN stimulation to exclude the influence of them. Second, the severity of COPD was low, and it is possible that the experiments were conducted at the emphysema or inflammatory condition. We predicted that the doses of PPE and LPS used in the present study would be relatively low for rats, and if the appropriate doses were applied, a more fulminant condition might be produced in a short term. Finally, all experiments in this study were performed under anesthetized condition. Previous literature reported that the respiration phase pattern and swallowing reflex occurrence are different in anesthetized and unanesthetized (conscious) condition (Feroah et al., 2002). Although the species is different, the difference of the respiration phase pattern and swallowing reflex occurrence is also thought to be present in rats, and therefore it is necessary to investigate using conscious rats.

## 5 Conclusion

Our study suggests that changes in the duty cycle and swallowing-related muscle activity may increase the risk of dysphagia in COPD. These findings may produce a new focus to treat COPD patients with dysphagia. However, our experiments were performed under anesthesia. Therefore, further studies on conscious conditions are required to better understand the relationship between COPD and dysphagia.

## Data Availability

The original contributions presented in the study are included in the article/Supplementary Material, further inquiries can be directed to the corresponding author.
